# An Efficient Grid-Based Geocasting Scheme for Wireless Sensor Networks

**DOI:** 10.3390/s23052783

**Published:** 2023-03-03

**Authors:** Neng-Chung Wang, Ming-Fong Tsai, Chao-Yang Lee, Young-Long Chen, Shih-Hsun Wong

**Affiliations:** 1 Department of Computer Science and Information Engineering, National United University, Miaoli 360302, Taiwan; 2 Department of Electronic Engineering, National United University, Miaoli 360302, Taiwan; 3 Department of Computer Science and Information Engineering, National Yunlin University of Science and Technology, Douliou 640301, Taiwan; 4 Department of Computer Science and Information Engineering, National Taichung University of Science and Technology, Taichung 404336, Taiwan

**Keywords:** Fermat point, geocasting, grid-based, Internet of Things, wireless sensor network

## Abstract

In a wireless sensor network (WSN), geocasting is a location-based routing protocol used for data collection or information delivery. In geocasting, a target region usually contains many sensor nodes with limited battery capacity, and sensor nodes in multiple target regions need to transmit data to the sink. Therefore, how to use location information to construct an energy efficient geocasting path is a very important issue. FERMA is a geocasting scheme for WSNs based on Fermat points. In this paper, an efficient grid-based geocasting scheme for WSNs, which is called GB-FERMA, is proposed. The scheme uses the Fermat point theorem to search for the specific nodes as Fermat points in a grid-based WSN, and it selects the optimal relay nodes (gateways) in the grid structure to realize energy-aware forwarding. In the simulations, when the initial power 0.25 J, the average energy consumption of GB-FERMA is about 53% of FERMA-QL, 37% of FERMA, and 23% of GEAR; however, when with the initial power 0.5 J, the average energy consumption of GB-FERMA is about 77% of FERMA-QL, 65% of FERMA, and 43% of GEAR. The proposed GB-FERMA can effectively reduce the energy consumption and thus prolong the lifetime of the WSN.

## 1. Introduction

The wireless sensor network (WSN) is a very important research field for the technology of the Internet of Things (IoT) [1,2,3]. A wireless sensor network (WSN) consists of a large number of sensor nodes and a sink. Sensor nodes are deployed in a wide area for sensing the relevant information, and transmit the sensing data to the sink [4,5,6]. Recently, there has been a trend towards large-scale deployments with thousands and thousands of sensor nodes being deployed over a vast field. In this case, the sink communicates with other sensor nodes via the geographic information. As well as that, the WSNs often apply the many-to-one pattern. Thus, geocasting has been employed to data dissemination from a sink to geographically related multiple destinations [7]. Geocasting is a routing protocol that starts sending a query message from the sink to sensor nodes in one or multiple geographic target regions, and sensor nodes then periodically transmit sensing data to the sink [8].

The battery of sensors has the restrictions for continuously carrying out the tasks that are sensing information, transmitting packet, and forwarding messages. In a large-scale WSN environment, it is difficult for sensor nodes to charge or replace batteries, so how to effectively use power to prolong the lifetime of the WSN is an important issue [9]. In this paper, we focus on the problem of geocasting with multiple target regions in the large-scale WSN. We propose an efficient geocasting scheme with a grid-based shared tree path for WSNs, called GB-FERMA.

The contributions of the proposed GB-FERMA consist of three parts. First, an area of interest is defined as a target region, and then the data for each target region is collected into the region head. Second, the GB-FERMA constructs an efficient grid-based geocasting tree for data transmission. The grid-based shared tree path is constructed for multiple region heads and the sink. A cell head is elected from each cell for data sending, data forwarding, and data fusion, and then the cell head is used for data transmission in grid-based geocasting. Finally, GB-FERMA selects the optimal relay nodes (gateways) for data transmission, reducing the energy consumption of sensor nodes.

The rest of this paper is organized as follows. Section 2 introduces the related background of this work. In Section 3, we describe the proposed grid-based geocasting scheme for WSNs. Simulation results are presented in Section 4. Section 5 draws conclusions.

## 2. Related Work

A WSN usually consists of a large number of sensor nodes and a sink. In general, a sink has infinite power, whereas sensors are usually equipped with low-capacity batteries. Because sensors have limited power resources, researchers aim to build a power-saving algorithm and employ an energy efficient mechanism to extend network lifetime [10].

Recently, a lot of research has focused on the power-limited issue in WSN by using a grid-based structure. Chi and Chang [11] proposed an energy-aware grid-based routing scheme with mobile observers (EAGER) for WSN. In the scheme, a virtual grid structure is constructed and keeps the grid head to disseminate data. Other sensor nodes in a cell apply a time-scheduling method to sleep at specific periods for the purpose of energy saving. Regarding the routing maintenance process, EAGER presents a re-routing approach to reduce the overheads to decrease the energy utilization of routing construction. Khan et al. [12] proposed a virtual grid-based dynamic routes adjustment scheme (VGDRA). VGDRA aims to reduce the energy cost by minimizing the routing reconstruction. The communication rules applied to the data delivery route re-adjustment toward the mobile sink only requiring a limited number of sensor nodes. Meng et al. [13] proposed a grid-based reliable routing protocol (GBRR), which creates a virtual cluster based on a square grid. This is achieved by the intra-cluster and inter-cluster communication quality. The scheme calculates the best paths along the cluster to avoid the overload of head nodes. Fan et al. [14] proposed a grid-based multicast routing approach for WSNs. This approach achieves better overall performance through the concept of game balance in game theory. The scheme deals fairly with all factors, namely space, energy, and data factors to solve the multicast problem of the Nash Equilibrium.

There is also a significant amount of literature that conducts the geocasting problem in WSNs, and the application often needs to collect or monitor the information in several location-based regions [15]. Yu et al. [16] designed a geographical and energy-aware routing protocol (GEAR) to disseminate the packet from regions. GEAR utilizes a recursive geographical forwarding strategy by using energy-aware and geographically-informed neighbor selection. Y.-M. Song et al. [17] proposed an efficient geocasting protocol for WSNs with multiple target regions, called FERMA. FERMA utilizes the characteristics of Fermat point [18,19] to construct the shortest path for data transmission and minimize energy consumption. This scheme constructs a shared path among multiple target regions via Fermat points to decrease the cost of packet forwarding. S. Park et al. [20] developed a mobile geocasting protocol (M-Geocasting) to deal with the data delivery issue between the regions and the mobile sink. The mobile sink has geographically collective mobility, and M-Geocasting provides the location information of a sink group to all sensor nodes in the target regions. Wang et al. [21] proposed energy-efficient Q-learning-based geocasting (FERMA-QL) for WSNs, where the nodes closest to the Fermat points become relay nodes, and then Q-learning is used to construct a shared path for further data transmission. Varun and Gangwar [22] proposed a geometrical link-aware geocasting scheme with energy balancing (GeoLE) for WSNs. GeoLE selects different groups of relay sensors to balance the energy consumption of the sensors according to the predictive relay selection (PRS) metric. The PRS metric contains the transmission count, forwarding progress, delay, and remaining energy. Ghosh [23] explored the impact of mobility on the energy consumption and transmission delay for Wireless Adhoc and Sensor Networks (WASNs). The proposed protocol uses the Fermat point-based routing with mobility assisted to reduce energy consumption and transmission delay in WASNs. In this paper, we propose an efficient grid-based geocasting scheme (GB-FERMA) for WSNs. We use a grid structure in the large-scale WSN to improve the performance of FERMA.

## 3. The Proposed Scheme

The geocasting focuses on location-based routing. This aims to solve the routing issue that is available in more than one region in the network to propose an efficient grid-based geocasting scheme. If there is only one region target needed to transmit data, that is a simple and normal problem, and the shortest path can be established to deal with that.

### 3.1. System Model

In this study, the network field is divided into *M* × *N* virtual cells of grid, where *M* and *N* are both positive integers. Each cell is a square with cell size α, so the area of the cell is α × α. The cell size $\alpha =R_{tr}/\left(2\sqrt{2}\right)$ is calculated by using the transmission range $R_{tr}$. Any node in a cell can communicate directly with others nodes in its neighboring cells. In the proposed scheme, a pair of numbers is used to identify each cell, called grid identification (GID). The GID of each cell is [*CX*, *CY*], and the GIDs of cells in the first row are [1, 1], [2, 1], [3, 1], [4, 1], and [5, 1] from left to right. Similarly, the GIDs of cells in the second row are [1, 2], [2, 2], [3, 2], [4, 2], and [5, 2], and so on.

### 3.2. Cell Head Election

**Figure 1:**
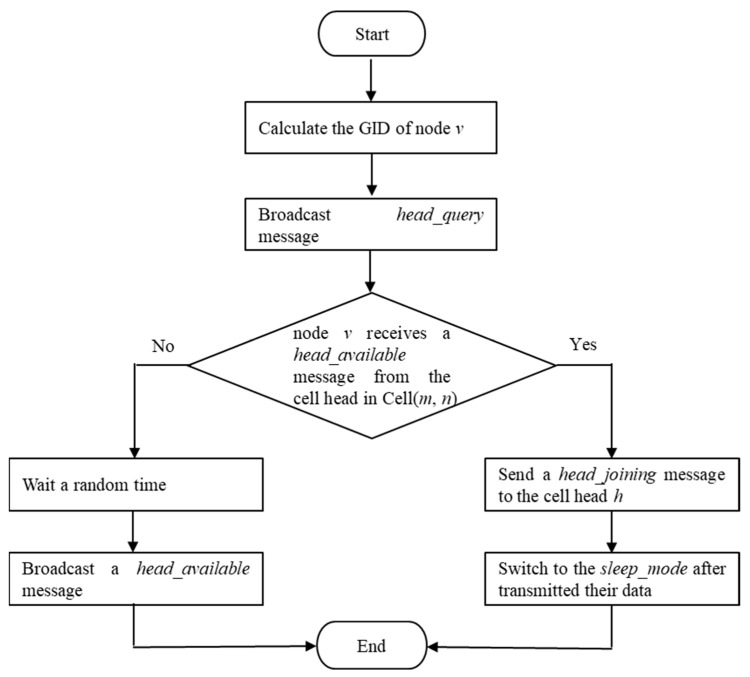
Flowchart of Cell Head Election Algorithm.

In a cell, there may be a lot of sensor nodes available. A cell head is the node that is elected in the cell for data sending, data forwarding, and data fusion. This study uses the Cell Head Election Algorithm, as shown in Algorithm 1, for cell head election in a cell. If a node *v* is available, in line 1, it calculates the GID of node *v*, called Cell(*m*, *n*). In line 2, a node *v* must join a cell, and it broadcasts the *head_query* message to all neighbor nodes. The *head_query* message carries the information <*Type*, *SourceGID*, *GID*>. *Type* is the packet type of message. *SourceGID* is the GID of the source. In line 3, if node *v* receives a *head_available* message from the cell head in Cell(*m*, *n*), this state means there is a cell head available in Cell(*m*, *n*). The *head_available* message includes the information <*Type*, *HeadID*, *GID*>. *HeadID* is the GID of the cell head. In line 4, node *v* joins the cell head *h* in Cell(*m*, *n*), and sends the *head_joining* message. In line 5, for the power saving, node *v* switches to the sleep mode after transmitting its data. The *head_joining* message consists of the information <*Type*, *SourceID*, *GID*>. At system initialization, all the sensors have a uniform battery level, and no cell head is available in a cell. Therefore, node *v* cannot receive a *head_available* message from the cell head in Cell(*m*, *n*). In lines 8 and 9, it waits a random time, and then broadcasts a *head_available* message. Due to the CSMA/CA method in the IEEE 802.11 based WLAN standard, a node transmits the sensing data only when the channel is sensed to be idle. Thus, if a node in the Cell(*m*, *n*) is the first to broadcast a *head_available* message, it is the cell head in Cell(*m*, *n*). The flowchart of Cell Head Election Algorithm is shown in Figure 1.
**Algorithm 1:** Cell Head Election Algorithm//Cell head election algorithm at node *v* in Cell(*m*, *n*) 1:  Calculate the GID of node *v*. 2:  Broadcast *head_query* message. 3:  **if** (node *v* receives a *head_available* message from the cell head in Cell(*m*, *n*)) {. 4:   Send a *head_joining* message to the cell head *h*. 5:   Switch to the *sleep_mode* after transmitted their data. 6:  } 7:  **else** { 8:   Wait a random time 9:   Broadcast a *head_available* message 10: }

In a cell, there is only one node that is a cell head to carry out the task for information sensing, forwarding messages, and data fusion. The others are all sleeping, and awakened if necessary.

### 3.3. Region Construction and Region Head Election

**Figure 2:**
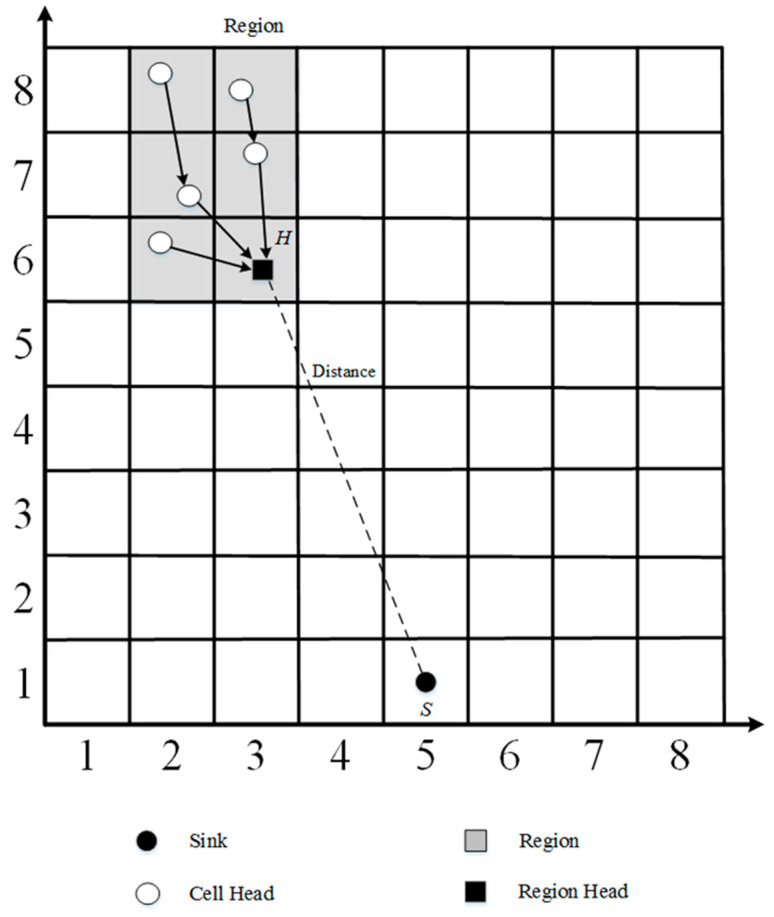
Region head election.

The geocasting scheme is used for disseminating tactical messages to nodes within a specific area. This study constructs target regions firstly and then elects a region head in each target region. The sink defines an area of interest as a target region and includes one or more cell, which is calculated by using the location and the GID. As shown in Figure 2, in a target region, it also needs a region head *H* for carrying out the data collection and data transmission. The region head *H* of the target region is the node that has the minimum distance between the sink and the nodes in the target region. This study uses the MAODV algorithm [24] to construct a shortest path tree at the region head *H*.

### 3.4. Gateway Selection

**Figure 3:**
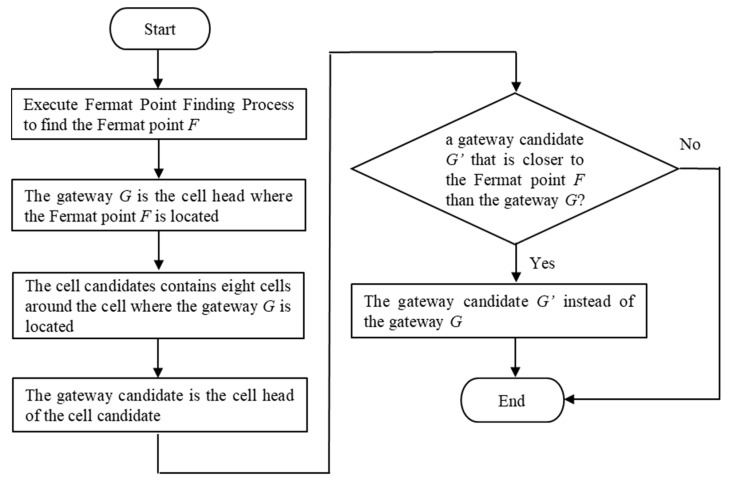
Flowchart of Gateway Selection Algorithm.

Geocasting refers to the delivery of data from a group of destinations identified by locations. The sink assigns one or more location areas as the target regions, and collects the sensing data from these target regions to the sink. However, the sensors are usually power limited. This work aims to construct the shortest path tree to achieve the energy-saving objective via Fermat points. The Fermat point is decided using the Fermat point theorem [17]. The Fermat point is characterized by the smallest sum of distances from the three vertices of the triangle. This Fermat point in any triangle can be found by the Fermat Point Finding Process, as shown in Process 1. The Gateway Selection Algorithm is shown in Algorithm 2 and the flowchart of the Gateway Selection Algorithm is shown in Figure 3.
**Process 1:** Fermat Point Finding Process**Step 1:** At any edges in △*ABC*, we can construct three regular triangles: △*A’BC*, △*AB’C*, and △*ABC’*. **Step 2:** The Fermat point is the intersection point of the three straight line segments: $\bar{AA’}$, $\bar{BB’}$, and $\bar{CC’}$.

**Algorithm 2:** Gateway Selection Algorithm//Suppose there are two target region heads and the sink: 1:  Execute Fermat Point Finding Process to find the Fermat point *F*; 2:  The gateway *G* is the cell head where the Fermat point *F* is located; 3:  The cell candidates contain eight cells around the cell where the gateway *G* is located; 4:  The gateway candidate is the cell head of the cell candidate; 5:  **if** (a gateway candidate *G’* that is closer to the Fermat point *F* than the gateway *G*); 6:   The gateway candidate *G’* instead of the gateway G.

**Figure 4:**
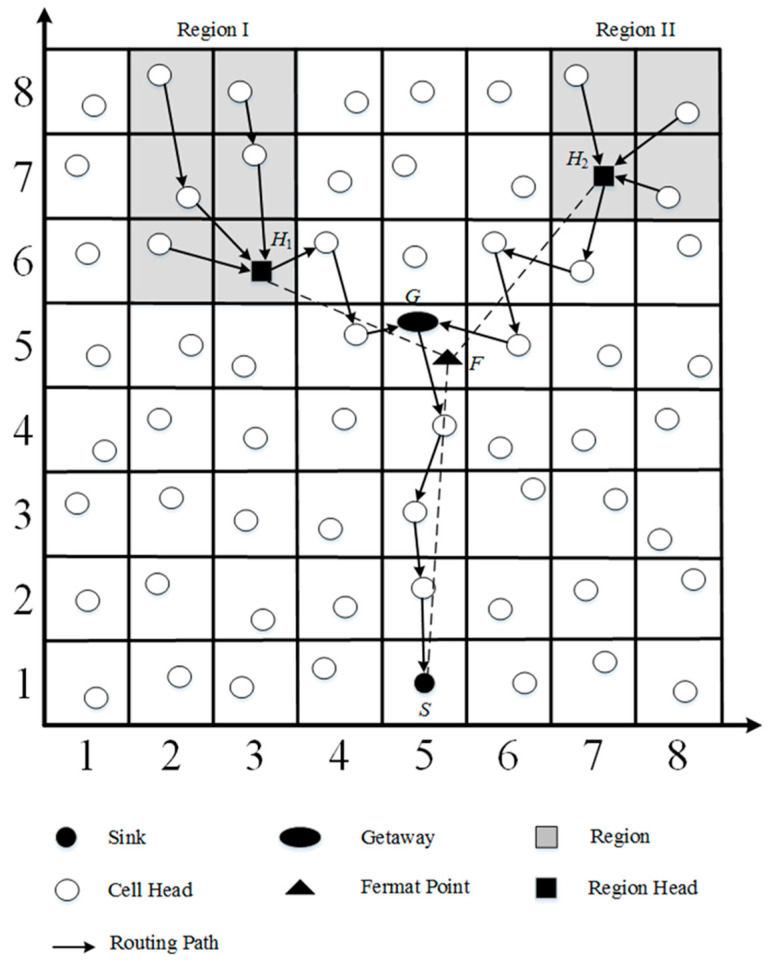
Gateway selection.

We give an example to illustrate gateway selection, as shown in Figure 4. In the example, there are two target regions in the network area. After the region head is determined, the sink *S* calculates the Fermat point *F* and its GID. In this work, the cell head where the Fermat point is located is defined as gateway *G*. The purpose of gateway *G* is to merge the information from *H*_1_ and *H*_2_, and forward the data to the sink.

In general, the geocasting could be constructed alone the line segments $\bar{H_{1}G}$, $\bar{H_{2}G}$, and $\bar{SG}$. In the mathematical view, the geocasting along the line segments $\bar{H_{1}G}$, $\bar{H_{2}G}$, and $\bar{SG}$ is the shortest path tree. However, in the grid-based network, this topology is not the best one due to the fact that any cell head can communicate directly with neighboring cell heads. The gateway of the cell where Fermat point *F* is located will cause the redundant path. Thus, the location of the gateway should be adjusted. If gateway *G’* is closer to the Fermat point *F* than gateway *G*, gateway *G’* is then instead gateway *G*.

**Figure 5:**
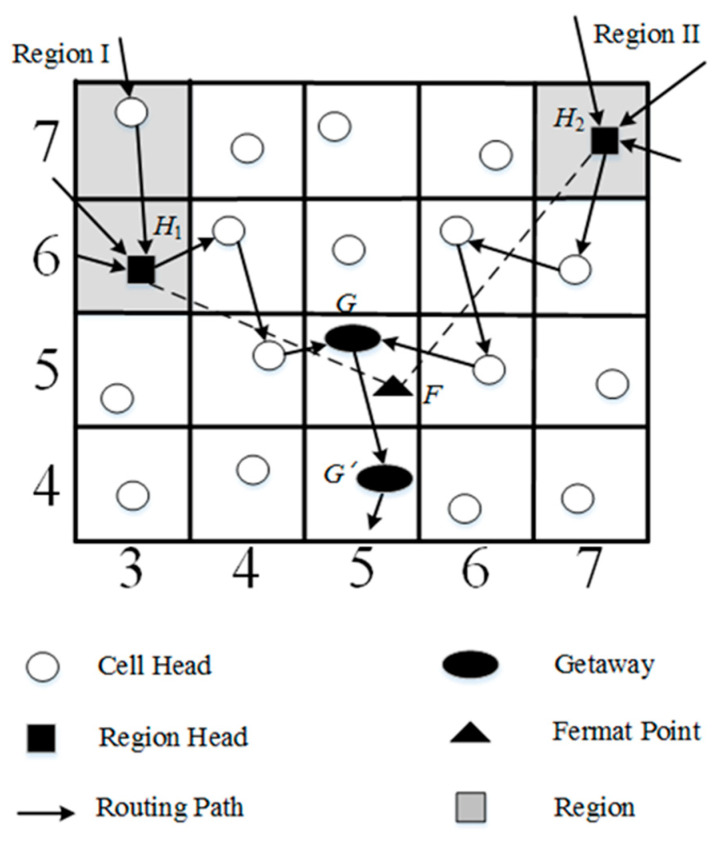
Gateway adjustment.

The cell candidate set is the set of cells where the gateway candidates are located. As shown in Figure 5, there are eight cells around the cell where gateway *G* is located, and cell candidate set θ is <[4, 6], [5, 6], [6, 6], [4, 5], [6, 5], [4, 4], [5, 4], [6, 4]>. The cell candidate set is along the line segment $\bar{H_{1}G}$ and the line segments $\bar{H_{2}G}$ are $\rho_{1}$ and $\rho_{2}$, respectively. The available cell candidate set μ can then be determined by Equation (1).

(1) $$\mu =\theta \;-(\rho_{1}\cup \rho_{2})$$

We suppose the available gateway candidate set is *S_agc_* for the available cell candidate set μ. Gateway $G^{\prime }$ can then be determined by Equation (2).

(2) $$G^{\prime }=\{\;p\;|\;p=p_{i}\;\mathrm{with}\;\mathrm{minimum}\;d\left(p_{i},\;s\right),\;\mathrm{where}\;p_{i}\in S_{agc}\;\mathrm{and}\;1\leq i\leq n\;\}$$

where $\;d\left(p_{i},\;s\right)$ is the distance between the location of each available gateway candidate $\;p_{i}$, the sink is *s*, and the number of members in *S_agc_* is *n*.

### 3.5. Energy Efficient Grid-Based Geocasting Path Construction

After the region head selection process and gateway selection process, the sink can construct the energy efficient grid-based geocasting path between the sink and the two target regions. The operations for route discovery are as follows. First, when the sink calculates the location of the region head and gateway, it floods a route request (RREQ) packet on the network with the time to live (TTL) value towards gateway *G*. The RREQ packet carries the information <*Type*, *SourceGID*, *DestGID*, *PathTravel*, *TTL*> in its header. The *SourceGID* and *DestGID* refer to the GID of the source and destination, respectively. The *PathTraversed* records the routing information. In this case, the sink broadcasts the RREQ packet, and its information is <*RREQ*, *Sink GID*, *Gateway GID*, *PathTravel*, *TTL*>.

**Figure 6:**
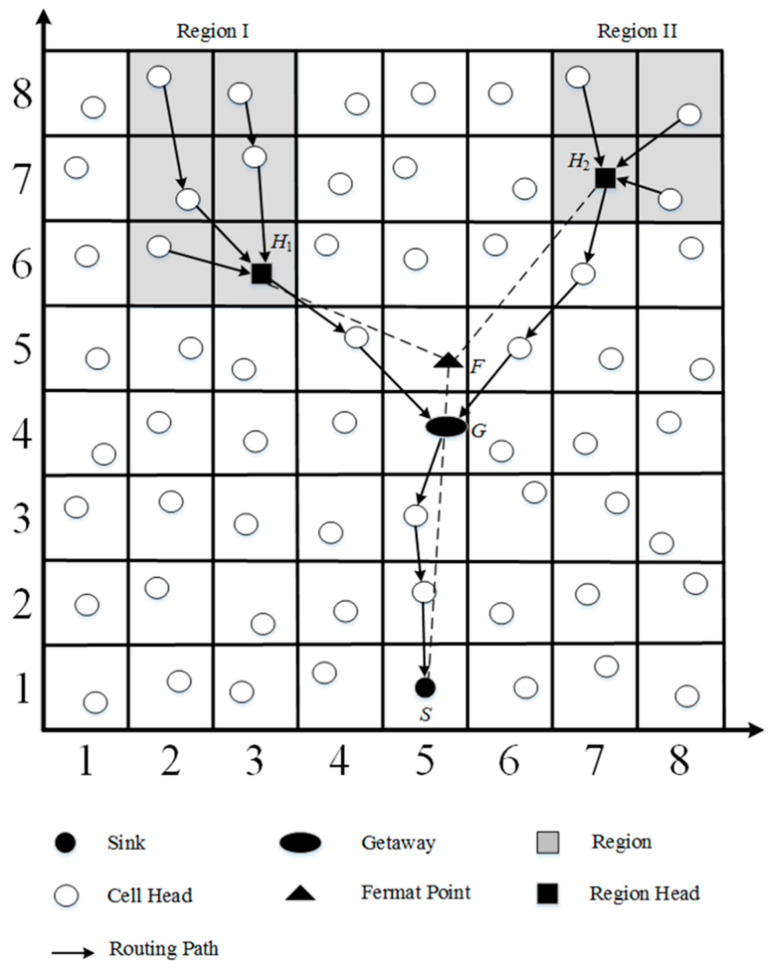
Energy efficient grid-based geocasting path construction.

When any cell header receives a RREQ packet, it first checks the *DestGID* in the RREQ to determine whether the entry is its own address. If not, this node appends its GID into the *PathTravel* and decreases the value of *TTL* by one. It then floods the RREQ packets on the network until the value of *TTL* is zero. When the gateway *G* receives a RREQ packet, it floods two RREQ packets on the network to the region heads *H*_1_ and *H*_2_ and sends a route reply (RREP) packet back to the sink, respectively. The RREP packet includes the information <*Type*, *SourceGID*, *DestGID*, *ReversePath*, *TTL*>. Here the *Type* is RREP and *ReversePath* and includes the reverse path from *PathTravel* of RREQ. Gateway *G* also tries to find the shortest path routes from the gateway to the two region heads, *H*_1_ and *H*_2_. When a region head (*H*_1_ or *H*_2_) receives a RREQ packet, it then sends a RREP packet back to the gateway. Finally, an energy efficient grid-based geocasting path could be constructed, as shown in Figure 6.

### 3.6. Region Head Joining

**Figure 7:**
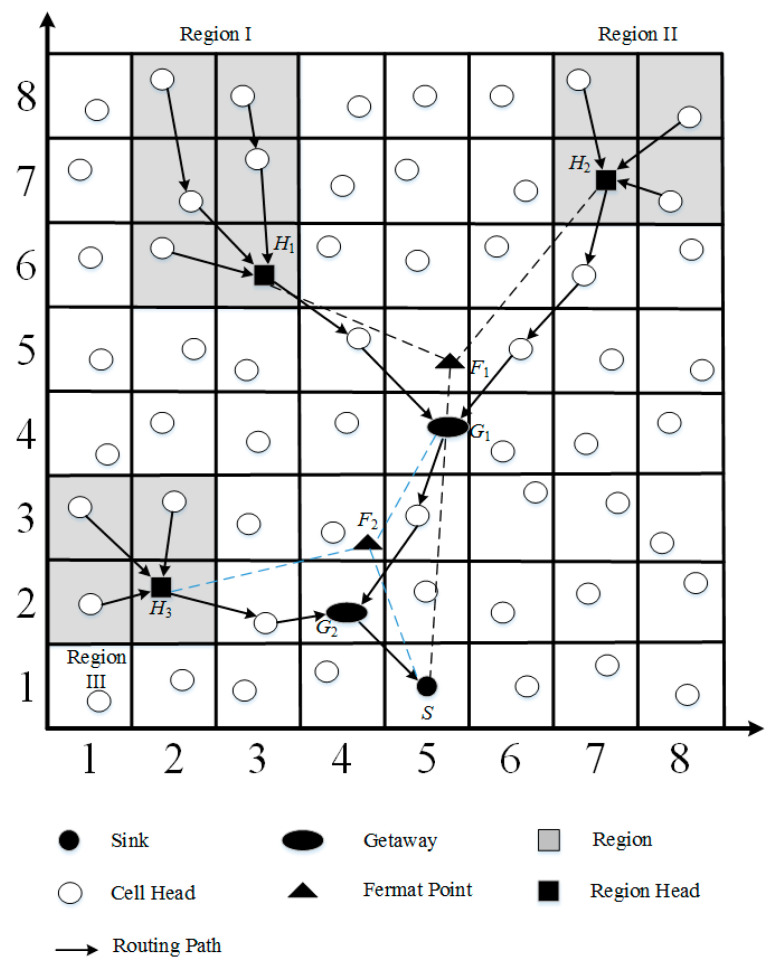
Region head joining.

In the proposed GB-FERMA, we use Fermat points to find the shortest path to construct an energy efficient path for grid-based geocasting. If there are more than two target regions needed to collect data, the GB-FERMA constructs a tree structure by selecting the two region heads farthest from the sink. As shown in Figure 7, the gateway *G*_1_ could be found by using the gateway selection process. A new region III could then be joined into the tree-based transmission path. Gateway *G*_1_ and the region head *H*_3_ conduct the gateway selection process, and then an energy efficient tree-based shortest path can be constructed.

## 4. Simulation Results

In this section, we performed simulations to compare the performance of the proposed GB-FERMA with that of FERMA-QL [21], FERMA [17], and GEAR [16]. These schemes are implemented in MATLAB for simulations. The number of sensor nodes is increased from 200 to 1200, and the number of sensor nodes is increased by 200 each time. The sensor nodes are randomly deployed with the initial power of 0.25 J and 0.5 J in a 200 m × 200 m network area. The number of cells is 10 m × 10 m. We assume that the sink is located at (0, 0) with an unlimited power supply. Each packet size is 2000 bits.

In the simulations, the energy model adapts the First Order Radio Model [25]. The energy consumption of the transmitting node is determined by Equation (3) and the energy consumption of the receiving node is determined by Equation (4). These two equations define the energy consumption of *k*-bit packets to transmit data over distance *d*. *E_elec_* is the energy consumed by a sensor node used in a transmitter or a receiver circuit. *E_amp_* is the energy consumed by the amplifier used to send data. In addition, the transmitted data have a *d^2^* energy loss over distance *d*.

*E_Tx_*(*k*, *d*) = *E_elec_* × *k* + *E_amp_* × *k* × *d*^2^ (3)

*E_Rx_*(*k*) = *E_elec_* × *k* (4)

### 4.1. Average Energy Consumed versus Number of Target Regions

**Figure 8:**
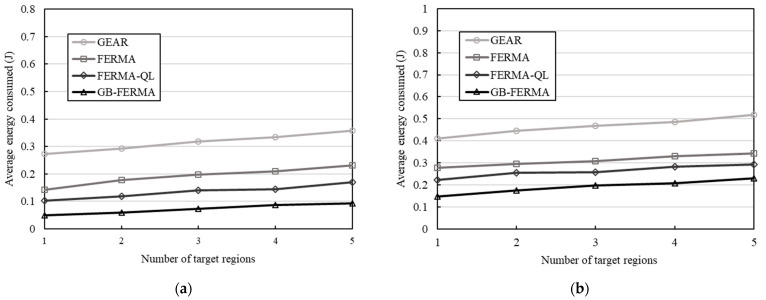
Average energy consumed versus number of target regions: (**a**) initial power 0.25 J; (**b**) initial power 0.5 J.

We first explored the average energy consumed under a different number of target regions. The number of sensor nodes is 200 nodes, the initial power of sensor nodes is 0.25 J and 0.5 J, and the number of cells is 10 × 10. As shown in Figure 8, we change the number of target regions and observe the average energy consumption when using GEAR, FERMA, FERMA-QL, and GB-FERMA, respectively. GEAR sends the sensing data for individual areas of interest. FERMA uses the Fermat points to find the nearest relay nodes (gateways) to gather data from the target regions and forwards it to the sink. FERMA-QL is a variant version of FERMA based on Q-learning. GB-FERMA applies a grid structure and calculates Fermat points to find optimal relay nodes (gateways) with the shortest path. It is responsible for aggregating the data from the target regions and transmitting the data to the sink. When the initial power is 0.25 J, the average energy consumption of GB-FERMA is about 53% of FERMA-QL, 37% of FERMA, and 23% of GEAR; however, when with the initial power 0.5 J, the average energy consumption of GB-FERMA is about 77% of FERMA-QL, 65% of FERMA, and 43% of GEAR. The average energy consumption of GB-FERMA is lower than that of GEAR and FERMA, which can effectively extend the network lifetime.

### 4.2. Number of Rounds versus Number of Nodes

**Figure 9:**
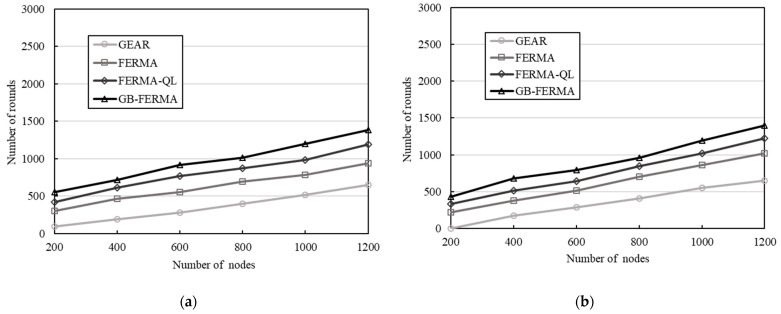
Number of rounds versus number of nodes: (**a**) initial power 0.25 J; (**b**) initial power 0.5 J.

Next, we discuss the influence of the impact of the changes in the number of nodes. The number of nodes starts from 200 nodes and increases by 200 each time up to 1200 nodes. In Figure 9, the number of rounds for transmission that the WSN performed is shown under various numbers of nodes. In the simulation, as the number of nodes increases, the number of rounds that can be performed by each scheme also increases. Among them, the number of rounds performed by the proposed GB-FERMA is the best. For 1200 sensor nodes with two different initial power levels, the number of rounds performed by GB-FERMA is about 1.2 times of FERMA-QL, 1.5 times of FERMA, and 2.1 times of GEAR.

### 4.3. Number of Alive Nodes versus Number of Rounds

**Figure 10:**
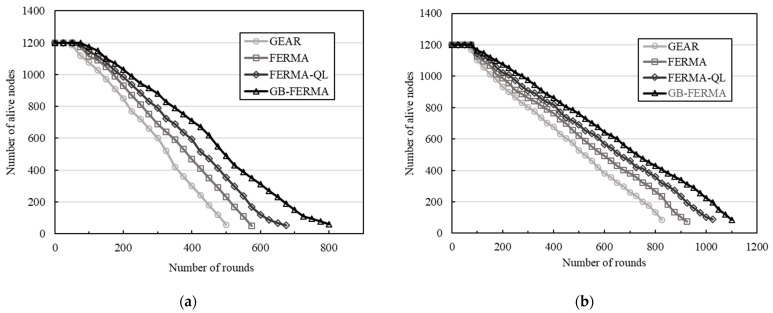
Number of alive nodes versus number of rounds: (**a**) initial power 0.25 J; (**b**) initial power 0.5 J.

In this work, the network lifetime is defined as the number of rounds before the sensor nodes are unable to transmit sensing data to the sink. Figure 10 shows the network lifetime performance for the three geocasting schemes. In this simulation, the number of nodes is 1200 and the target region is 5. With the initial power 0.25 J, the number of transmission rounds is about 800 for GB-FERMA, 675 for FERMA-QL, 575 for FERMA, and 500 for GEAR; however, with the initial power 0.5 J, the number of transmission rounds is about 1100 for GB-FERMA, 1025 for FERMA-QL, 925 for FERMA, and 825 for GEAR. Simulation results show that GB-FERMA can effectively increase the number of surviving nodes, thereby prolonging the network lifetime.

### 4.4. Total Energy Consumed versus Number of Rounds

**Figure 11:**
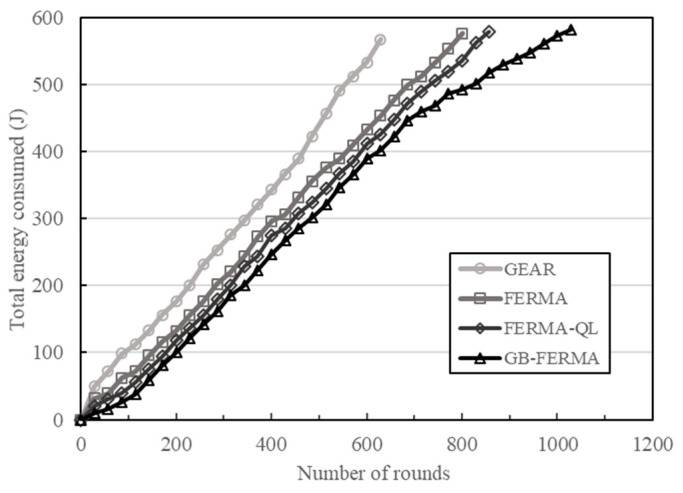
Total energy consumed versus number of rounds.

We observe the total energy consumed under various numbers of rounds. As shown in Figure 11, the proposed GB-FERMA has less total energy consumption compared to FERMA-QL, FERMA, and GEAR. The GB-FERMA uses the grid-based data aggregation tree to transmit data, which can effectively reduce energy consumption. It is proved that GB-FERMA can extend the lifetime of WSN more than the other three schemes.

The comparisons of GB-FERMA, FERMA-QL, FERMA, and GEAR are shown in Table 1. GB-FERMA is a geocasting scheme that is grid based; while FERMA-QL, FERMA, and PEDAP are geocasting schemes without being grid based. The data transmission of GB-FERMA, FERMA-QL, and FERMA is the greedy routing with optimal relay nodes, while the data transmission of GEAR is the greedy routing. The routing path types of GB-FERMA, FERMA-QL, FERMA, and GEAR are a grid-based shared tree path, Q-learning based shared tree path, shared tree path, and chain path, respectively. The energy performance of GB-FERMA is the best among the four schemes.

## 5. Conclusions

In this study, an efficient grid-based geocasting scheme called GB-FERMA is proposed in WSNs. GB-FERMA was developed to deal with the geocasting through a grid-based structure and Fermat points theory. GB-FERMA selects the optimal relay nodes (gateways) via the Fermat points calculation based on the Fermat point theorem to achieve energy efficient data transmission. In the simulations, when the initial power is 0.25 J, the average energy consumption of GB-FERMA is about 53% of FERMA-QL, 37% of FERMA, and 23% of GEAR; however, when with the initial power is 0.5 J, the average energy consumption of GB-FERMA is about 77% of FERMA-QL, 65% of FERMA, and 43% of GEAR. The proposed GB-FERMA is more capable of reducing energy consumption and prolonging the lifetime of WSNs than FERMA-QL, FERMA, and GEAR.

## Figures and Tables

**Table 1:** The comparisons of GB-FERMA, FERMA-QL, FERMA, and GEAR.

Protocol	GB-FERMA	FERMA-QL	FERMA	GEAR
Geocasting with grid-based	Yes	No	No	No
Strategy of data transmission	Greedy routing with optimal relay nodes	Greedy routing with optimal relay nodes	Greedy routing with optimal relay nodes	Greedy routing
Type of routing path	Grid-based shared tree path	Q-learning based shared tree path	Shared tree path	Chain path
Energy performance	Excellent	Great	Good	General

## Data Availability

Not applicable.
